# Structural Basis of Brr2-Prp8 Interactions and Implications for U5 snRNP Biogenesis and the Spliceosome Active Site

**DOI:** 10.1016/j.str.2013.04.017

**Published:** 2013-06-04

**Authors:** Thi Hoang Duong Nguyen, Jade Li, Wojciech P. Galej, Hiroyuki Oshikane, Andrew J. Newman, Kiyoshi Nagai

**Affiliations:** 1 MRC Laboratory of Molecular Biology, Francis Crick Avenue, Cambridge CB2 0QH, UK

## Abstract

The U5 small nuclear ribonucleoprotein particle (snRNP) helicase Brr2 disrupts the U4/U6 small nuclear RNA (snRNA) duplex and allows U6 snRNA to engage in an intricate RNA network at the active center of the spliceosome. Here, we present the structure of yeast Brr2 in complex with the Jab1/MPN domain of Prp8, which stimulates Brr2 activity. Contrary to previous reports, our crystal structure and mutagenesis data show that the Jab1/MPN domain binds exclusively to the N-terminal helicase cassette. The residues in the Jab1/MPN domain, whose mutations in human Prp8 cause the degenerative eye disease retinitis pigmentosa, are found at or near the interface with Brr2, clarifying its molecular pathology. In the cytoplasm, Prp8 forms a precursor complex with U5 snRNA, seven Sm proteins, Snu114, and Aar2, but after nuclear import, Brr2 replaces Aar2 to form mature U5 snRNP. Our structure explains why Aar2 and Brr2 are mutually exclusive and provides important insights into the assembly of U5 snRNP.

## Highlights

• We report the structure of Brr2 helicase in complex with the Jab1/MPN domain of Prp8
• Retinitis pigmentosa mutations in the Jab1/MPN domain of Prp8 disrupt this complex
• Mechanism is proposed for the U4/U6 snRNA duplex unwinding and spliceosome activation
• The Brr2-Jab1/MPN and Aar2-Prp8 complexes provide insight into U5 snRNP biogenesis

## Graphical Abstract

**Figure:**
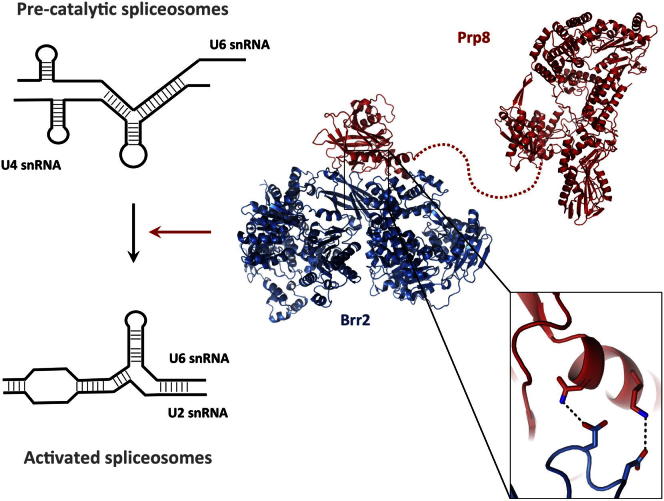

## Teaser

Brr2 helicase unwinds the U4/U6 snRNA duplex and introduces U6 snRNA into the active site cavity of the spliceosome formed within Prp8. Nguyen et al. report a crystal structure of Brr2 helicase together with the Jab1/MPN domain of Prp8 and provide new insights into this process as well as retinitis pigmentosa.

## Introduction

The spliceosome is a dynamic molecular machine that catalyzes the excision of introns from premessenger RNA (pre-mRNA) and ligation of exons in a two-step *trans*-esterification reaction. It comprises five RNA-protein subunits, namely U1, U2, U4, U5, and U6 small nuclear ribonucleoprotein particles (snRNPs), and various non-snRNP factors, which assemble on pre-mRNA substrates in a highly ordered manner (Wahl et al., 2009). After the binding of U1 snRNP to the 5′ splice site (5′-SS) and U2 snRNP to the branch point (BP) in the pre-mRNA, the remaining three snRNPs join the complex as U4/U6.U5 tri-snRNP. Three U5 snRNP proteins, the DExD/H-box family helicase Brr2, the EF2-like GTPase Snu114, and Prp8, play crucial roles in the activation of the spliceosome and the formation of the catalytic core for the two *trans*-esterification reactions (Wahl et al., 2009). The spliceosome undergoes major structural and compositional changes, including the separation of the U4/U6 snRNA duplex catalyzed by Brr2 (Laggerbauer et al., 1998; Raghunathan and Guthrie 1998; Makarov et al., 2002) and concomitant formation of the catalytic center, consisting of a highly structured RNA network between U2, U5, and U6 snRNAs and the 5′-SS and BP sequences of the pre-mRNA (Will and Lührmann, 2011). Snu114 regulates the activity of Brr2 in a nucleotide-dependent manner (Small et al., 2006; Bartels et al., 2003). Prp8 accommodates crucial small nuclear RNAs (snRNAs) and substrate residues—U5 snRNA, U6 snRNA, 5′-SS, BP, and 3′-SS—within a cavity revealed recently by structural analysis (Galej et al., 2013; Turner et al., 2006). After lariat introns and mature mRNA are released from the spliceosome, U2, U5, and U6 snRNPs are recycled for the next round of splicing, and Brr2 also plays a role in this process (Small et al., 2006).

Brr2 is one of the largest proteins (246 kDa in *Saccharomyces cerevisiae*) and the only Ski2-like helicase in the spliceosome (Bleichert and Baserga, 2007). The Brr2 sequence showed an N-terminal region (∼470 amino acids) of unknown function and two consecutive helicase cassettes, each containing a helicase core with two RecA domains followed by a Sec63 unit. The structure of the C-terminal Sec63 unit of yeast Brr2 revealed two helical domains followed by a fibronectin3-like (FN3) domain (Pena et al., 2009; Zhang et al., 2009), where the helical domains resemble the ratchet and helix-loop-helix domains (fourth and fifth domains) of the DNA helicase Hel308, despite the low sequence identity (Büttner et al., 2007). Alignment of Brr2 with Hel308 indicated the presence of a winged helix domain (WH) as the third of six domains in each helicase cassette of Brr2. The structure of human Brr2 reported recently confirmed this domain organization (Santos et al., 2012). The N-terminal helicase cassette with high sequence similarity to the conserved motifs of the DExH-box RNA helicase family has been shown to be functional and essential in yeast for cell viability and the unwinding activity of Brr2 (Kim and Rossi, 1999). The C-terminal cassette deviates more from the consensus helicase motifs and lacks residues important for ATP hydrolysis. The intercassette interactions have been extensively investigated, and the C-terminal cassette was proposed to regulate the activity of the N-terminal cassette (Santos et al., 2012).

A salt-stable complex consisting of Brr2, Prp8, and Snu114 can be isolated from human U5 snRNP (Achsel et al., 1998), and these three proteins interact functionally and genetically (van Nues and Beggs 2001; Liu et al., 2006). The Jab1/MPN domain from the C terminus of Prp8 (Pena et al., 2007; Zhang et al., 2007) forms a complex with Brr2 and strongly stimulates its helicase activity (Maeder et al., 2009; Mozaffari-Jovin et al., 2012). The degenerative eye disease retinitis pigmentosa type 13 (RP13) is caused by mutations in the Jab1/MPN domain of human Prp8 (McKie et al., 2001). Mutations of equivalent residues in the yeast Jab1/MPN domain disrupt its interaction with Brr2 (Maeder et al., 2009). In this study, we determined the crystal structure of yeast Brr2 in complex with the Jab1/MPN domain of Prp8 to define their interaction surface, and structure-guided mutagenesis confirmed that the observed contacts are responsible for stimulating Brr2′s helicase activity. In the cytoplasm, Prp8 forms a precursor complex with U5 snRNA, seven Sm proteins, Snu114, and the U5 assembly factor Aar2, but after nuclear import, Brr2 replaces Aar2 to form mature U5 snRNP (Boon et al., 2007; Weber et al., 2011). We recently showed that Aar2 organizes the large reverse transcriptase (RT)-like and endonuclease (En)-like domain (Galej et al., 2013) and RNaseH-like (Pena et al., 2008; Ritchie et al., 2008; Yang et al. 2008) and Jab1/MPN domains (Pena et al., 2007; Zhang et al., 2007) of Prp8 into a stable assembly (Galej et al., 2013). By combining the analysis of the Prp8-Aar2 and Brr2-Jab1/MPN complexes, we are able to explain why Brr2 and Aar2 are mutually exclusive. Therefore, our Brr2-Jab1/MPN structure provides important insight into the molecular pathology of RP13, U5 snRNP biogenesis, and the formation of the active site of the spliceosome.

## Results

### Overall Structure

**Figure 1:**
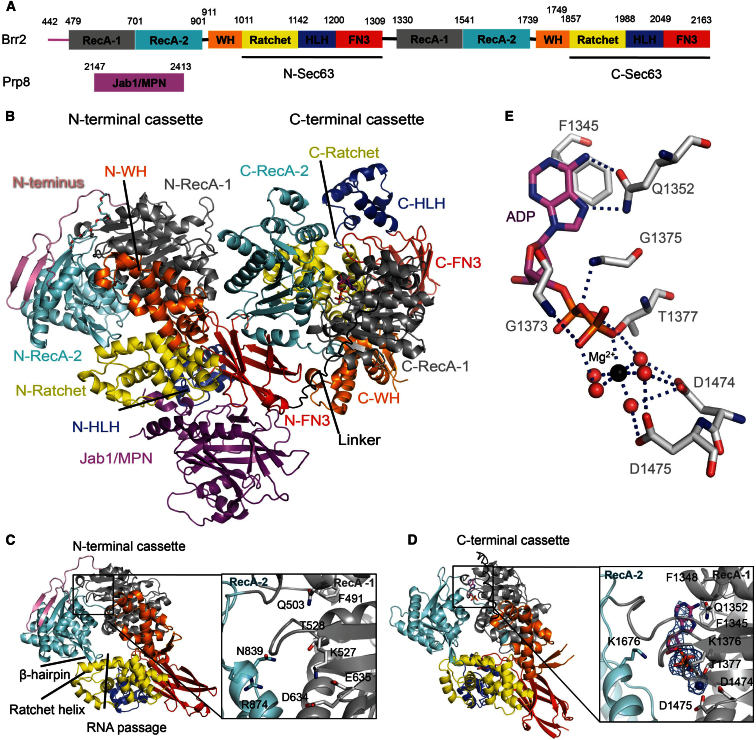
Structure of Yeast Brr2 in Complex with the Jab1/MPN Domain of Prp8 (A) Domain architecture of yeast Brr2 and the Jab1/MPN domain of Prp8. (B) Overview of the Brr2-Jab1/MPN domain complex. Domains are color-coded: pink, N-terminal extension; gray, RecA-1; cyan, RecA-2; orange, WH; yellow, ratchet; navy, HLH; red, FN3 domains. See also Figure S1. (C) N-terminal helicase cassette of Brr2 with inset showing the ATPase active site with no bound ADP. (D) C-terminal helicase cassette with inset showing the defective ATPase site with bound Mg^2+^-ADP. The *F*_*O*_*-F*_*C*_ difference map (sharpened with B = −48 Å^2^) calculated prior to including the Mg^2+^-ADP is shown as blue mesh and contoured at 3.0σ. (E) The interaction between the β-phosphate of ADP and two aspartates (D1474 and D1475) is mediated by a hydrated Mg^2+^ ion. The Mg^2+^ ion is bound by five water molecules and an oxygen atom from the β-phosphate of the ADP molecule in an octahedral arrangement. See also Figures S2 and S3.

Yeast Brr2 truncated at the N terminus (residues 442–2163) was crystallized together with the Jab1/MPN domain of yeast Prp8 (residues 2147–2413). Experimental phases were obtained to 4.5 Å resolution by single isomorphous replacement anomalous scattering (SIRAS) using a K_2_OsO_4_ derivative and extended to 3.1 Å of the native amplitudes by density modification with inclusion of the molecular replacement solutions of the Jab1/MPN domain (Pena et al., 2007) and the second Sec63 unit of Brr2 as partial models (Pena et al., 2009; Zhang et al., 2009; see Experimental Procedures and Figures S1A and S1B available online). The structure has been refined at 3.1 Å resolution to R_work_/R_free_ of 21.4%/27.0% with excellent stereochemistry (Figures S1C and S1D; Table 1). The yeast Brr2 (yBrr2) in the Jab1/MPN domain complex is similar in architecture to the free human Brr2 (hBrr2) with Cα root-mean-square deviation of 2.8 Å for 1,545 aligned residues out of 1,707 residues of yBrr2, both consisting of two structurally similar helicase cassettes forming a compact entity (Figures 1 and S2A–S2D; Santos et al., 2012). Each helicase cassette is composed of six domains: two RecA domains followed by WH, ratchet, helix-loop-helix (HLH), and FN3 domains (Figure 1A); the latter three domains are collectively referred to as a Sec63 unit (Pena et al., 2009; Zhang et al., 2009). In the N-terminal cassette of yBrr2 residues 442–478 preceding the RecA-1 domain form two β strands joined to the β sheet in the RecA-2 domain (Figure 1B), as in hBrr2. Both helicase cassettes are architecturally similar to Hel308 helicase (Büttner et al., 2007), except that Hel308 lacks FN3. The RecA-1, RecA-2, WH, and ratchet domains are packed in a circular arrangement, forming a passage that in Hel308 has been shown to accommodate the 3′-single strand of partially unwound duplex DNA (Figure 1C; Büttner et al., 2007). A prominent β-hairpin (residues 851–862 in the N-terminal cassette) extending from RecA-2 toward the ratchet domain corresponds to the Hel308 β-hairpin that separates the two strands of DNA. The FN3 domain is interposed between the ratchet and HLH domains and interacts with both. In the yBrr2-Jab1/MPN complex, however, the bulk of the N-terminal HLH domain (residues 1150–1195) may be mobile, showing *B*-factors twice the molecular average, while a loop (residues 1826–1840) in the C-terminal WH domain showed no electron density. The HLH domain interacts with the FN3 domain and hangs outside the ring formed by the first four domains (Figures 1C and 1D). The C-terminal cassette lacks the conserved motifs required by DExH-box helicases for ATP hydrolysis and has been shown to be inactive (Figures S2E, S2F, and S3; Kim and Rossi, 1999; Santos et al., 2012). However, in the C-terminal cassette, there is good density for Mg^2+^-ADP bound to the Q-loop (residues 1348–1356) and motif I (residues 1372–1377) (Figures 1D and 1E), whereas in the N-terminal cassette, no bound nucleotide was observed (Figure 1C). In human Brr2 crystals grown from a high salt condition, no nucleotide binding was observed (Santos et al., 2012). However, after stabilization of crystals by cross-linking with glutaraldehyde and transfer to a low salt solution containing ATP, ATP was observed in the C-terminal cassette, whereas ADP was observed in the N-terminal cassette (Santos et al., 2012). No ADP was added, so the authors inferred that the ADP bound to the N-terminal cassette came from ADP contaminants in the ATP. It is not clear whether the observed difference in nucleotide binding between human and yeast proteins is functionally significant, because different conditions were involved.

### The Interface between Brr2 and the Jab1/MPN Domain of Prp8

**Figure 2:**
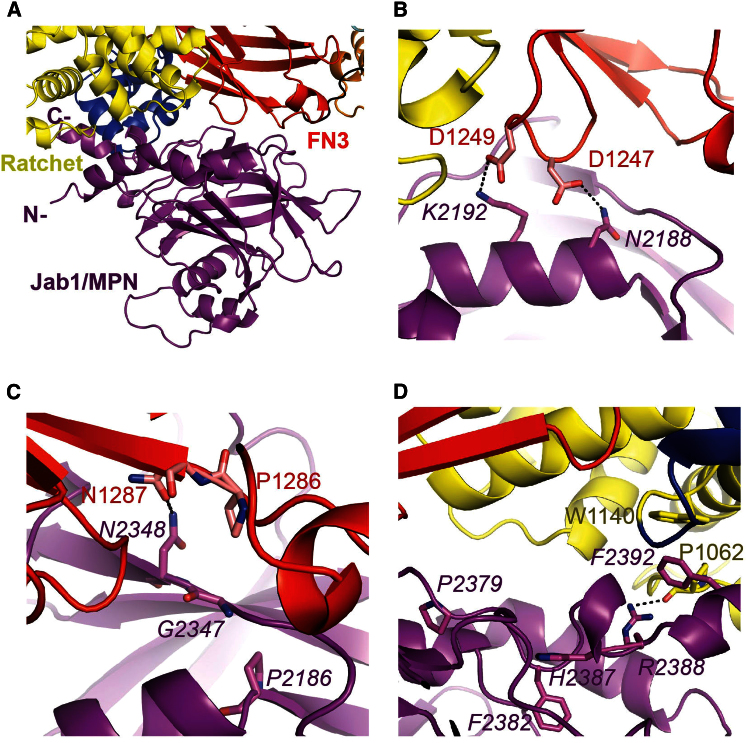
Interface between Brr2 and the Jab1/MPN Domain of Prp8 (A) Close-up view of the Jab1/MPN domain and its interface with Brr2. (B) Intersubunit salt-bridge and hydrogen bond involving D1247 and D1249 in the fibronectin-3 domain of Brr2. (C) Location of *prp8-1* mutant (*G2347D*) and important neighboring residues in the Jab1/MPN domain and Brr2. (D) Residues in the Jab1/MPN domain in yeast Prp8 corresponding to residues in human counterpart mutated in retinitis pigmentosa type 13 and key residues for interaction in Brr2. See also Figures S3 and S4 and Tables S1 and S2.

The Jab1/MPN domain (residues 2147–2413) of Prp8 interacts with the ratchet and FN3 domains of the N-terminal Sec63 unit (residues 1011–1309), burying 1,536 Å^2^ of solvent-accessible surface (Figures 2A–2D and S4A–S4C; Table S1). The N- and C-terminal helices of the Jab1/MPN domain are gathered to the same side that covers the bottom face of the Sec63 unit. Residues in the Jab1/MPN domain are in italics hereafter. In all Jab1/MPN domain structures determined so far, the residues corresponding to yeast *N2384–R2388* form a 3_10_ helix or a turn, and the C-terminal polypeptide displays an extended conformation (Pena et al., 2007; Zhang et al., 2007; Galej et al., 2013; Figure S5A). In contrast, in our Brr2-Jab1/MPN complex structure, the C-terminal tail folds into a helical conformation at the interface with the ratchet and HLH domains (Figure 2A). The last 18 residues (*S2396*–*S2413*) of Jab1/MPN are not visible in our structure or in the previous structures (Pena et al., 2007; Zhang et al., 2007; Galej et al., 2013).

**Figure 3:**
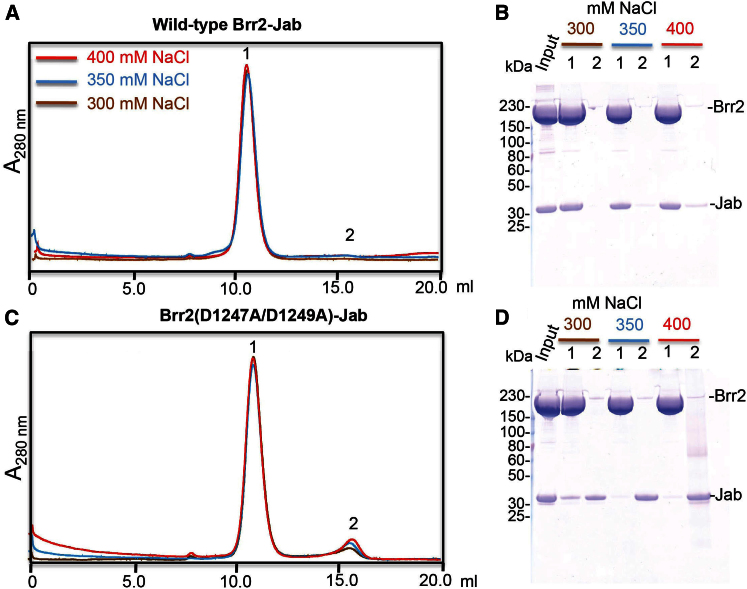
Effects of Brr2 Double Mutation D1247A/D1249A on Jab1/MPN Interaction (A) Size exclusion chromatography analysis of 1:1 mixtures of wild-type Brr2 and the Jab1/MPN domain of Prp8 at 300 (brown trace), 350 (blue trace), and 400 mM (red trace) NaCl. (B) SDS-PAGE analysis of the protein composition in peaks 1 and 2 for each salt concentration. (C and D) Similar to (A) and (B), respectively, except analyses were performed on 1:1 mixtures of the double mutant D1247A/D1249A and the Jab1/MPN domain.

**Figure 4:**
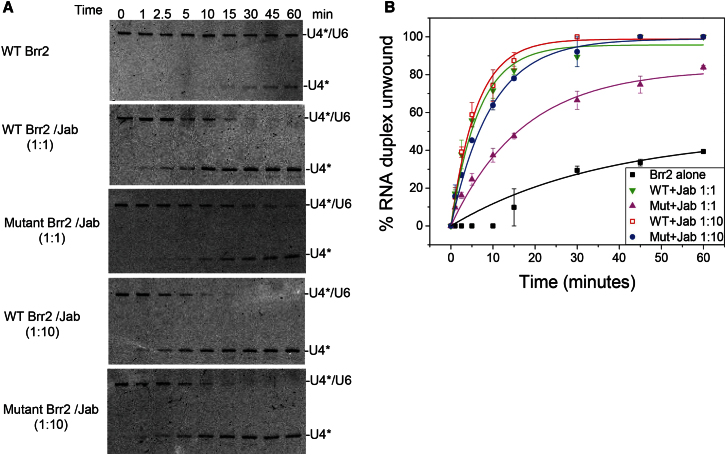
Effects of Brr2 Double Mutation D1247A/D1249A on Unwinding Activity (A) Unwinding time course comparing the unwinding enhancement of wild-type and mutant Brr2 by the Jab1/MPN domain toward U4/U6 duplex. Each unwinding reaction (10 μl) was initiated by addition of 2 mM ATP in 30 mM Tris HCl pH 8.0, 60 mM NaCl, 2.5 mM MgCl_2_, 1.5 mM DTT, and 100 ng/μl acetylated bovine serum albumin. Concentrations: 40 nM, U4^*^/U6 (the asterisk denotes labeled RNA); 20 nM, Brr2. WT, wild-type. (B) Unwinding time course was fitted to an exponential equation: % duplex unwound = A[1 − exp(−*k*_u_t)], where *k*_u_ is the apparent unwinding rate and A is the amplitude. Errors were obtained from two independent experiments for each time course. A(wild-type Brr2) = 48 ± 4, *k*_u_ = 0.028 ± 0.005 min^−1^; A(wild-type Brr2+Jab 1:1) = 96 ± 3, *k*_u_ = 0.15 ± 0.02 min^−1^; A(mutant Brr2+ Jab 1:1) = 83 ± 3, *k*_u_ = 0.057 ± 0.007 min^−1^; A(wild-type Brr2+ Jab 1:10) = 99 ± 2, *k*_u_ = 0.16 ± 0.02 min^−1^; A(mutant Brr2 + Jab 1:10) = 99 ± 2, *k*_u_ = 0.105 ± 0.009 min^−1^.

Yeast two-hybrid assays (Liu et al., 2006; Pena et al., 2007) indicated that the Jab1/MPN domain binds to the C-terminal helicase cassette, which is inconsistent with our crystallographic results. Therefore, we mutated the highly conserved residues D1247 and D1249 in the N-terminal FN3 domain; these residues form a hydrogen bond with *N2188* and a salt-bridge with *K2192* of the Jab1/MPN domain, respectively (Figure 2B). The interaction between the Jab1/MPN domain and the wild-type or the D1247A/D1249A mutant of Brr2 was investigated by size-exclusion chromatography in three different salt concentrations, and the protein samples of peak fractions were analyzed by SDS-PAGE. The 1:1 mixture of wild-type Brr2-Jab1/MPN was eluted as a single peak, which contained both Brr2 and the Jab1/MPN domain for all the salt concentrations (Figures 3A and 3B), whereas for the D1247A/D1249A mutant Brr2 mixture, a second peak was observed and became more prominent with increasing salt concentration (Figures 3C and 3D). Above 350 mM NaCl, the larger peak contained only Brr2 and the smaller peak contained the Jab1/MPN domain, showing that the D1247A/D1249A mutations substantially weakened Jab1/MPN binding. Although there is a crystal contact between the Jab1/MPN domain and the C-terminal Sec63 unit (Figure S5B), we observed no binding between the Jab1/MPN domain and C-terminal Sec63 unit in vitro (Figure S4D). These results led us to conclude that the interaction between Brr2 and the Jab1/MPN domain observed in our crystal is genuine. Previous yeast two-hybrid assays were carried out using a small fragment (*2239*–*2335*) of the Jab1/MPN domain (Pena et al., 2007) that would not form a stable structure (Figure S5C). It has been shown that the Jab1/MPN domain enhances the unwinding activity of Brr2 (Maeder et al., 2009; Mozaffari-Jovin et al., 2012). The D1247A/D1249A mutant of Brr2 had a lower unwinding activity than the wild-type in the presence of an equimolar amount of the Jab1/MPN domain; however, the addition of 10-fold excess of the Jab1/MPN domain restored the wild-type activity, showing that the double mutant of Brr2 has reduced affinity for the Jab1/MPN domain, but these mutations do not affect the intrinsic unwinding activity of Brr2 (Figure 4).

Our structure provides insights into the temperature-sensitive impairment of Brr2 stimulation by the *prp8-1* (*G2347D*) allele (van Nues and Beggs, 2001). The Asp side chain at *G2347* would point into the hydrophobic interior of Jab1/MPN and its energy cost—rising with temperature—can be alleviated only by a backbone rotation to point the Asp side chain outward (Figure 2C), which would then clash with P1286. This would prevent favorable interactions of the neighboring *T2346* and *N2348* with residues in two FN3 loops, including a *N2348*–N1287 hydrogen bond.

Mutations responsible for RP13 are found toward the C terminus of the Jab1/MPN domain (McKie et al., 2001; Pena et al., 2007; Zhang et al., 2007) and have been shown to weaken the interaction between Brr2 and the Jab1/MPN domain of Prp8 (Boon et al., 2007; Maeder et al., 2009). These residues are conserved between yeast and human (Table S2). *R2388* and *F2392* (*R2310* and *F2314* in human) are part of the interface with the ratchet domain. The *R2388K* mutation would weaken the hydrogen bond between the guanidinium side chain and the main chain carbonyl oxygen of P1062, while *R2388G* abolishes it (Figure 2D; Table S1). *F2392L* would eliminate stacking with W1140. *P2379, F2382*, and *H2387* (*P2301, F2304,* and *H2309* in human) are not in direct contact with Brr2 but provide a scaffold for the region around *I2378* and *E2385*, which are at the Brr2 interface (Figure 2D). The structure accounts for the reduced binding of these pathological mutant Jab1/MPN domains to Brr2 (Maeder et al., 2009), and therefore, it provides detailed insight into the molecular pathology of RP13.

### Biogenesis of U5 snRNP

**Figure 5:**
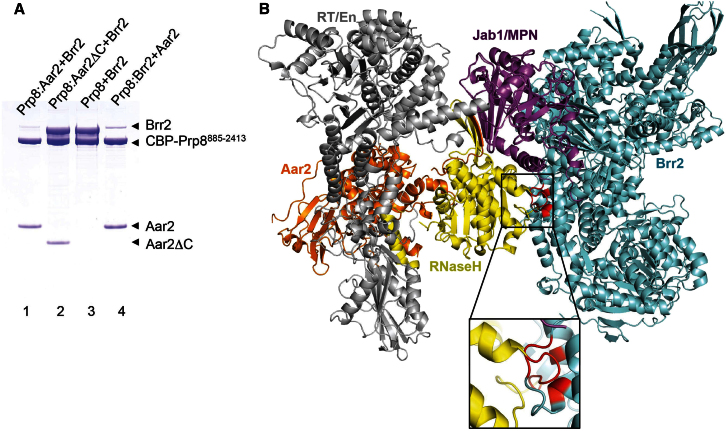
Interaction of Prp8 with Aar2 and Brr2 (A) SDS-PAGE of eluted peak fractions from four calmodulin pull-down experiments using a CBP at the N terminus of Prp8^885–2413^. The input proteins are labeled at the top of the gel. Brr2 fails to bind to the binary complex between Prp8 and Aar2 (lane 1). When the C terminus of Aar2 is deleted, Brr2 is able to bind the Prp8-Aar2ΔC complex (lane 2). Brr2 binds to Prp8 when Aar2 is removed from Prp8 (lane 3). Addition of Aar2 to Prp8-bound Brr2 results in the replacement of Brr2 upon prolonged incubation (lane 4). (B) Superposition of the Jab1/MPN domain (magenta) in the Brr2-Jab1/MPN and Prp8-Aar2 structures. The clash between the RNaseH-like domain (yellow) and Brr2 (cyan), which prevents Brr2 from binding to Prp8-Aar2 complex, is highlighted in red. Aar2 is shown in orange. See also Figures S5 and S6.

In yeast cytoplasm, Prp8 forms a complex with U5 snRNP assembly factor Aar2. After nuclear import of U5 snRNP precursors, Aar2 is replaced by Brr2 by a mechanism that was shown to involve phosphorylation of Aar2 (Weber et al., 2011). We carried out in vitro pull-down assays using Prp8^885–2413^ tagged with a calmodulin-binding peptide (CBP) at the N terminus. Brr2 fails to bind to the Prp8^885–2413^-Aar2 complex, and conversely, Aar2 fails to bind to the Brr2-Prp8^885–2413^ complex but replaces Brr2 after prolonged incubation (Figure 5A). Hence, the binding of Aar2 and Brr2 to Prp8^885–2413^ is mutually exclusive. Superposition of the Jab1/MPN domain in Brr2-Jab1/MPN and Prp8^885–2413^-Aar2 complex structures revealed a steric clash between the RNaseH-like domain of Prp8 and Brr2, which accounts for the mutual exclusivity of Aar2 and Brr2 (Galej et al., 2013; Figure 5B). In the Prp8^885–2413^-Aar2 complex, the Jab1/MPN domain is fixed to the RT/En domain mainly by the formation of a continuous β sheet with the C-terminal tail of Aar2 and the β-finger of the RNaseH-like domain (Galej et al., 2013; Figure S5D). When this C-terminal β strand of Aar2 is deleted, the Jab1/MPN domain is freed to interact with Brr2, producing a Prp8^885–2413^-Aar2ΔC-Brr2 ternary complex (Figure 5A). Our results provide a structural explanation why Brr2 and Aar2 are mutually exclusive.

## Discussion

The crystal structure of yBrr2 in complex with the Jab1/MPN domain has revealed the structural basis of the interaction between Prp8 and Brr2, which plays crucial roles in the activation of the spliceosome and formation of the active site. The Jab1/MPN domain of Prp8 interacts exclusively with the ratchet and FN3 domains in the N-terminal helicase cassette of Brr2, and the residues in the Jab1/MPN domain, whose mutations cause type 13 retinitis pigmentosa in human, are located at or near the interface. The structure of the complex shows how the RP13 mutations could disrupt the Prp8-Brr2 interaction.

The Jab1/MPN domain of Prp8 increases the unwinding activity of Brr2 in vitro. Crystal structure of Hel308 in complex with partially unwound duplex DNA substrate provides important insights into the Brr2 function (Protein Data Bank [PDB] ID: 2P6R; Büttner et al., 2007). In Hel308-DNA complex, the terminal base pair of the substrate DNA duplex is packed against the tip of the strand separator β-hairpin extending from the RecA-2 domain toward the ratchet domain; the emergent 3′-strand travels through the channel formed by the RecA1, WH, and ratchet domains and bends around the ratchet domain (Figure S6A), with its phosphate backbone making contact with the conserved Arg-Ala-Arg motif of the HLH domain. Deletion of the HLH domain or mutation of Arg662 within the conserved motif to Ala substantially increases the unwinding activity of Hel308 helicase (Richards et al., 2008), and the authors proposed that the HLH domain might act as an autoinhibitory domain or molecular brake. Structural superposition of Hel308 on the N-terminal cassette of yBrr2 verified a strong structural similarity, although the cleft between the RecA-2, ratchet, and HLH domains is wider and the strand-separator β-hairpin correspondingly longer in Brr2 (Figures S6A and S6B). Hel308 mutations that inhibit translocase activity map onto yBrr2 mutations that cause cold sensitivity and blocked splicing, such as *brr2-1* (E610G) in the RecA-1 domain and N1104L, R1107L, or R1107P in the putative ratchet helix (Pena et al., 2009; Zhang et al., 2009; van Nues and Beggs, 2001; Santos et al., 2012). One possible mechanism how the Jab1/MPN domain activates Brr2 could be releasing the brake imposed by the HLH domain. So far, no structure of Brr2 with an RNA substrate has been reported, but Santos et al. (2012) proposed that the 3′ strand of RNA may follow a similar path, because the mutation of two positively charged surface residues in the HLH domain, which do not contact other domains, strongly reduces U4/U6 snRNA duplex unwinding and the binding of RNA duplex with 31 nucleotide 3′ overhang. These results are not consistent with the autoinhibitory function of the HLH domain of Brr2.

Recently, we showed that Prp8 contains a large domain consisting of RT and En domains tightly associated through the linker (Ln) domain (Galej et al., 2013). The large RT/En domain is connected to the RNaseH-like domain via a short linker, whereas the RNaseH-like and Jab1/MPN domains are separated by a 70 residue unstructured spacer. Aar2 interacts with the large RT/En domain of Prp8 extensively across the junction between the Ln and En domains (Figure S5D). Its C-terminal tail (residues 348–353) forms a remarkable intermolecular, parallel β sheet with the β-hairpin of the RNaseH-like domain and the β-barrel of the Jab1/MPN domain, organizing the domains of Prp8 into a stable assembly. When the Jab1/MPN domain within the Brr2-Jab1/MPN complex is superimposed onto the Jab1/MPN domain within the Prp8-Aar2 complex, Brr2 and the RNaseH-like domain of Prp8 clash (Figure 5B, inset), showing why Brr2 is not able to bind Prp8 within the Prp8-Aar2 complex (Figure 5A, lane 1). Deletion of the C-terminal tail of Aar2 releases the Jab1/MPN domain from the tightly packed arrangement, allowing it to bind Brr2 (Figure 5A, lane 2). Likewise, in the absence of Aar2, Prp8 and Brr2 form a complex (Figure 5A, lane 3).

Hahn et al. (2012) recently showed that the N-terminal helicase cassette of Brr2 can be crosslinked in vivo to the conserved loop 1 of U5 snRNA and near the 5′-SS and 3′-splice site (3′-SS) of introns. Furthermore, the G858R mutation, which reduces the second-step splicing efficiency, enhances Brr2 crosslinking to 5′-SS, 3′-SS, and the BP regions, suggesting that these crosslinks are made in the active spliceosome immediately prior to step 2 of splicing (Hahn et al., 2012). Prp8 can likewise be crosslinked in vitro to loop 1 of U5 snRNA and to the 5′-SS, and BP(+2) nucleotides of pre-mRNA (Turner et al., 2006). Indeed BP(+2) nucleotide can crosslink to Prp8 in spliceosomes stalled before catalytic step 2 (A.J.N and C. M. Norman, unpublished data); this crosslink maps between residues 1585 and 1598 within a disordered loop in the active site cavity (Galej et al., 2013). These results together show that the N-terminal cassette of Brr2 lies in close proximity to the active site cavity of Prp8 prior to the second catalytic step. Our crystal structures suggest how the N-terminal cassette of Brr2 might be brought into close proximity with the active site cavity. Galej et al. (2013) showed that the majority of U4cs-1 and *brr2-1* suppressors (Kuhn and Brow, 2000; Kuhn et al., 1999) mapped on one face of the RT/En domain of Prp8, which could therefore form an additional interaction surface for Brr2. Brr2 placed adjacent to the active site cavity of Prp8 could introduce U6 snRNA unwound from U4 snRNA directly into the active site cavity with its translocase activity (Figure S6C). The RNaseH-like domain attached to the RT/En domain at the N-terminal end is linked at the C-terminal end to the Brr2-bound Jab1/MPN domain via a 70 residue linker. The position of the RNaseH-like domain could be modulated in response to conformational changes associated with the two catalytic steps of splicing (Query and Konarska, 2004; Yang et al., 2008).

The structure of Brr2 in complex with the Jab1/MPN domain of Prp8 has given us important insights into the mechanism of U5 snRNP biogenesis and the molecular pathology of RP13 mutations. Together with the close association of Brr2 with the 5′ and 3′ splice sites and U5 snRNA loop 1 revealed by the recent crosslinking results (Hahn et al., 2012), the structures of Brr2 and Prp8 (Galej et al., 2013) reveal how these crucial U5 snRNP proteins collaborate to shape the active site of the spliceosome.

## Experimental Procedures

### Protein Expression and Purification

Full-length Brr2 with a C-terminal His_8_ tag was expressed in *Saccharomyces cerevisiae* essentially as described previously (Galej et al., 2013). Cells were harvested by centrifugation and resuspended in lysis buffer (50 mM Tris HCl pH 8.0, 700 mM NaCl, 4 mM CaCl_2_, 2 mM Mg(OAc)_2_, 2 mM imidazole, 20 mM β-mercaptoethanol, 0.2% Igepal CA-630, and protease inhibitor cocktails [Roche]). The cells were frozen in liquid nitrogen and lysed in solid phase by Freezer Mill 6870 (SPEX CertiPrep). After centrifugation, the supernatant was incubated with Ni-nitrilotriacetic acid (NTA) agarose (QIAGEN) overnight at 4°C, washed with Ni500W buffer (20 mM Tris HCl pH 8.0, 500 mM NaCl, 20 mM imidazole, and 10 mM β-mercaptoethanol), and eluted with Ni500E buffer (Ni500W buffer supplemented with 250 mM imidazole). The His_8_-tag of the protein purified from the Ni-NTA agarose was cleaved by His-tagged tobacco etch virus (TEV) protease before passing through the Ni-NTA agarose again to remove His_8_ tag, uncleaved protein, and TEV protease. The protein collected in the flowthrough of the second Ni-NTA column was dialyzed against B220 buffer (20 mM 4-(2-hydroxyethyl)-1-piperazineethanesulfonic acid [HEPES] Na^+^ pH 7.5, 220 mM NaCl, and 10 mM β-mercaptoethanol). The protein was further purified on a HiTrap heparin column (GE Healthcare) and a MonoQ column (GE Healthcare) equilibrated with B220 buffer using NaCl gradient from 220 mM to 1 M. The protein was dialyzed against storage buffer containing 20 mM HEPES Na^+^ pH 7.5, 300 mM NaCl, 10 mM β-mercaptoethanol, and 5% glycerol. Purified Brr2 was incubated with chymotrypsin at a 1:500 w/w ratio for 1 hr at 25°C. Two millimolar phenylmethylsulfonyl fluoride was added to stop the proteolysis. The mixture was subjected to size-exclusion chromatography (Superdex 200, GE Healthcare) in storage buffer. Fractions containing the desired 200 kDa fragment were pooled and concentrated to 15–20 mg/ml for crystallization.

Prp8^2147–2413^ (Jab1/MPN) and Prp8^1806–2413^ (Prp8-CTR, RNaseH-Jab1/MPN) with a N-terminal His_6_ tag were expressed using pET28a vector (Novagen) in *E. coli* strain BL21 (DE3) pLysS. The cells were grown in 2 × TY (16 g Bacto-tryptone, 10 g yeast extract, 5 g NaCl per liter) liquid medium supplemented with 50 μg/ml kanamycin and 33 μg/ml chloramphenicol at 37°C until OD_600_ = 0.5, and the temperature was reduced to 18°C. Protein expression was induced by addition of 0.5 mM isopropyl-β-D-1-thiogalactopyranoside at OD_600_ = 0.8. Cells were harvested by centrifugation and resuspended in lysis buffer (20 mM Tris HCl pH 8.0, 500 mM NaCl, 20 mM imidazole, 20 mM β-mercaptoethanol, and protease inhibitor cocktail [Roche]). The cells were lysed by sonication. After centrifugation, the supernatant was loaded on a Ni-NTA sepharose column pre-equilibrated with Ni500W buffer. The protein was eluted with a linear gradient of imidazole from 20 to 250 mM. The eluate was dialyzed against B150 buffer (20 mM HEPES Na^+^ pH 7.5, 150 mM NaCl, and 10 mM β-mercaptoethanol) and further purified on a HiTrap heparin column equilibrated with B150 buffer using a NaCl gradient from 150 mM to 1 M, followed by gel-filtration in 20 mM HEPES Na^+^ pH 7.5, 250 mM NaCl, and 10 mM β-mercaptoethanol. The eluate was concentrated and dialyzed against B150 buffer. For the final step of purification, the sample was loaded on a MonoQ column equilibrated with B150 buffer and eluted with a NaCl gradient from 150 mM to 1 M. The purified protein was concentrated to 34 mg/ml in the same storage buffer as Brr2 for crystallization.

### Crystallization and Structure Determination

Proteolyzed Brr2 (15–20 mg/ml) was mixed with Prp8^2147–2413^ (34 mg/ml) in a 1:1.2 molar ratio and ADP to a final concentration of 2 mM and incubated on ice for 1 hr. Crystals of the complex were grown by sitting drop vapor diffusion at 20°C by mixing equal volumes (1 to 2 μl) of protein and reservoir solution (100 mM HEPES Na^+^ pH 7.5, 200 mM MgCl_2_, 32%–40% polyethylene glycol 400 [PEG400], and 6–12 mM cysteine). Crystal quality was improved by streak seeding after equilibrating the drop against the reservoir solution for 2 hr. Crystals appeared after 1 day and reached full size within 4 to 5 days. Crystals were incubated in cryobuffer (100 mM HEPES Na^+^ pH 7.5, 200 mM MgCl_2_, 25% PEG400, and 2 mM ADP) for 10–15 min prior to cryocooling by plunging into liquid nitrogen. For osmate derivatization, the crystals were first transferred to a fresh drop with cryobuffer for 30–60 min and then exchanged with the same buffer supplemented with 10 mM K_2_OsO_4_ overnight before cryocooling.

Diffraction data were collected at Diamond Light Source (beamlines I02 and I03) with a Pilatus 6 M detector. Native data were collected at 0.9795 Å wavelength and processed using iMosflm (Leslie and Powell, 2007) and AIMLESS (Evans, 2006). Native crystals diffracted anisotropically to 2.8–3.6 Å along different axes. The correlation between random half-sets of native intensity was 50% at 3.1 Å, and therefore, all data to 3.1 Å were included in structure determination. In order to obtain phases, single wavelength anomalous dispersion data from K_2_OsO_4_ derivatives were collected with very high redundancy at the midpoint between the peak and inflection energies (wavelength, 1.1402 Å) to reduce radiation damage and obtain anomalous and dispersion differences in one data set. We used low doses and fine-slicing (Pilatus detector) for better signal-to-background ratio. The data set that gave us phases was collected using 0.4° oscillation range, 1,200 images, 0.1 s exposure each, and 9% transmission and processed using XDS (Kabsch, 2010) and AIMLESS (Evans, 2006). We used the newer measure of data quality, CC(1/2) ≥ 0.5 (rather than I/sigma I), as the high resolution cutoff criterion (Karplus and Diederichs, 2012). Our refined model showed >93% correlation with Fobs, with >90% free correlation. PHASER (McCoy et al., 2007) was used to search for known fragments of Prp8^2147–2413^ (PDB: 2OG4) and Brr2^1851–2163^ (PDB: 3HIB) using resolution range 5–20 Å. The molecular replacement phases were used to find initial heavy atom sites by anomalous difference Fourier (fast Fourier transform) (Collaborative Computational Project, Number 4, 1994). The top three sites were refined, and further sites were found from residual map analysis in SHARP using the K_2_OsO_4_ data combined with the 3.1 Å native data (Vonrhein et al., 2007). The refinement was repeated in SHARP, and sites were added/removed from the results of each refinement. A total of eight sites were found and refined in SHARP, with phases calculated to 4.5 Å resolution. The overall figure of merit was 0.19 acentric and 0.20 centric. Phases were improved and extended to 3.1 Å resolution of the native amplitude by solvent flipping (Abrahams and Leslie, 1996), with the above molecular replacement solutions included as partial models in SHARP. The overall figure-of-merit was increased to 0.87. Automatic model building using ARP/wARP (Morris et al., 2003) implemented in SHARP further improved the map, which was used to start model building aided by optimally positioned homologous fragments. Hel308 from *Sulfolobus solfataricus* (PDB accession code: 2VA8; Richards et al., 2008) has the highest sequence identity to the first helicase cassette of Brr2 (19% sequence identity) and was selected as a search model. CHAINSAW (Collaborative Computational Project, Number 4, 1994) models (side chains truncated to gamma atoms) of Hel308 were made for the two helicase cassettes in Brr2 (to gamma atoms). MOLREP (Collaborative Computational Project, Number 4, 1994) was used to search for these models in the ARP/wARP (Morris et al., 2003) map using a resolution range of 5–20 Å. The N-terminal cassette was located readily with a contrast score of 5.98. The C-terminal cassette with 15% sequence identity could be located only by searching for two copies of the CHAINSAW model for that cassette. The first solution coincided with the N-terminal cassette already found, and the second solution gave the C-terminal cassette with a contrast score of 6.3. The location of Brr2^1851–2163^ (C-terminal Sec63 unit) by molecular replacement allowed us to distinguish the two cassettes unambiguously. Only the first RecA domain of the Hel308 model fitted the initial map; other domains required real-space rigid body refinement followed by extensive rebuilding. The structure was manually rebuilt in Coot (Emsley et al., 2010) and refined to 3.1 Å in Refmac5 under phase restraint of the SIRAS phases at 4.5 Å resolution (Murshudov et al., 2011). After the model was refined to R_work_/R_free_ = 24.3%/30.0%, the native amplitudes were corrected for anisotropy with truncation in the weak directions to Fσ F = 3 (http://www.doe-mbi.ucla.edu/sawaya/anisoscale/), and refinement continued until R_work_/R_free_ = 24.0%/28.4%. The resulting model was refined again using the native amplitudes to R_work_/R_free_ = 21.4%/27.0%. The final model contains residues 442–2163 of Brr2, excluding the disordered loop (1826–1840) in the C-terminal WH domain, and residues 2148–2395 of Prp8, plus one Mg^2+^-ADP molecule, one PEG400 molecule, and 15 bound water molecules. The model has excellent stereochemistry (MolProbity score in the 99^th^ percentile) and Ramachandran distribution (Table 1) for its resolution range. Buried solvent-accessible surface area was analyzed on the PDBePISA server (http://www.ebi.ac.uk/pdbe/). The structure illustrations were produced using Pymol (http://www.pymol.org).

### Calmodulin Pull-Down Assay

Various complexes of CBP-tagged Prp8^885-2413^ were used as baits in pull-down assay: (1) Prp8^885–2413^:Aar2; (2) Prp8^885–2413^:Aar2ΔC (missing 23 C-terminal residues); (3) Prp8^885–2413^; and (4) Prp8^885–2413^:Brr2. Samples were mixed with 2-fold excess of prey protein (Brr2 for baits 1–3 and Aar2 for bait 4) to final concentrations of 0.5 and 1.0 μM, respectively. The mixture was incubated at 4°C overnight in CALW buffer (20 mM HEPES K^+^ pH 7.8, 300 mM KCl, 1 mM Mg(OAc)_2_, 2 mM CaCl_2_, and 10 mM β-mercaptoethanol) and subsequently captured with calmodulin resin for 3 hr at 4°C. The resin was washed with CALW buffer, and proteins were eluted with CALE buffer (20 mM HEPES K^+^ pH 7.8, 300 mM KCl, 2 mM EGTA, and 10 mM β-mercaptoethanol).

### Gel-Filtration Analysis of Wild-Type/Mutant Brr2 and Jab1/MPN Domain

Wild-type or double mutant (D1247A/D1249A) Brr2 mixed with the Jab1/MPN domain at a 1:1 molar ratio (12 μM) was dialyzed against 20 mM HEPES Na^+^ pH 7.5, 10 mM β-mercaptoethanol with 300, 350, or 400 mM NaCl at 4°C. One hundred microliters of the mixture was loaded on a Superdex 200 10/300 GL column (GE Healthcare) pre-equilibrated with the same buffer. Peak fractions were concentrated and analyzed by SDS-PAGE for each NaCl concentration.

### Unwinding Assay

U4 snRNA was 3′-fluorescein-labeled and annealed to unlabeled U6 snRNA. Each unwinding reaction (10 μl) consisted of 40 nM U4^*^/U6 (^*^ denotes labeled RNA) and 20 nM Brr2 or Brr2-Jab1/MPN complex in 30 mM Tris HCl pH 8.0, 60 mM NaCl, 2.5 mM MgCl_2_, 1.5 mM dithiothreitol (DTT), and 100 ng/μl acetylated bovine serum albumin. The reaction mixture was incubated at room temperature for 5 min before initiation of the reaction by addition of 2 mM ATP and incubation at 30°C. At each time point, 1 μl was withdrawn and terminated with 6.5 μl of stop buffer containing 2% SDS, 0.4 M NaCl, and 40 mM EDTA. The samples were analyzed on 9% polyacrylamide native gel in 1 × Tris-borate-EDTA at 7 W for 2.5 hr at 4°C. The gel was visualized by a Typhoon imager and analyzed by ImageJ (http://rsbweb.nih.gov/ij/), and data were fitted using Origin (OriginLab).

## Figures and Tables

**Table 1:** Data Collection, Phasing, and Refinement Statistics

Data Set	Native	K_2_OsO_4_
Space group	P2_1_2_1_2_1_	P2_1_2_1_2_1_

**Cell Dimensions**		

*a*, *b*, *c* (Å)	107.6, 178.6, 180.4	108.5, 179.2, 180.4
α, β, γ (°)	90, 90, 90	90, 90, 90
Wavelength (Å)	0.9795	1.1402^a^
Resolution (Å)	92.2–3.1 (3.18–3.10)	127.1–4.5 (4.86–4.50)
*R*_sym_	0.075 (0.884)	0.34 (3.26)
*I*/σ*I*	9.7 (1.4)	8.9 (1.0)
Completeness (%)	99.9 (100)	100 (100)
Redundancy	4.2 (4.0)	9.3 (9.1)

**Phasing Method**		**SIRAS**

No. of sites		8
Acentric/centric mean figure of merit		0.19/0.20

**Refinement**		

Resolution (Å)	92.4–3.1 (3.18–3.10)	
No. reflections	60,299 (3,206)	
*R*_work_ (%)	21.4 (30.6)	
*R*_free_ (%)	27.0 (40.9)	
No. atoms	15,738	
Protein	15,668	
Ligand/ion	55	
Water	15	
*B*-factors (Å^2^)	122.6	
Protein	122.6	
Ligand/ion	130.4	
Water	88.4	

**Root-Mean-Square Deviations**		

Bond lengths (Å)	0.103	
Bond angles (°)	1.477	

**Ramachandran Statistics (%)**^b^		

Favored/allowed/outliers	93.48/6.11/0.41	

Values in parentheses are of the highest resolution shell.

a Data were collected at a wavelength that is at the midpoint between the peak and inflection wavelengths.

b Calculated using MolProbity (Davis et al., 2004).

## Accession Numbers

The coordinates and structural factors of the yeast Brr2^442–2163^-Prp8^2148–2395^ complex have been deposited in the PDB with the accession number 4BGD.

## Supplemental Information

Document S1. Supplemental Experimental Procedures, Figures S1–S6, and Tables S1 and S2

PDB Session S1. Crystal Structure of Yeast Brr2 in Complex with the Jab1/MPN Domain of Prp8, Related to Figure 1
