# Supplementary material for: Structural Basis of Brr2-Prp8 Interactions and Implications for U5 snRNP Biogenesis and the Spliceosome Active Site
Source: Structure. 2013 Jun 4;21(6):910–9. doi: 10.1016/j.str.2013.04.017 (PMC3677097; doi:10.1016/j.str.2013.04.017)
Supplement: Document S1. Supplemental Experimental Procedures, Figures S1–S6, and Tables S1 and S2 [file mmc1.pdf]

Structure, Volume *21*

Supplemental Information

Structural Basis of Brr2-Prp8 Interactions and Implications for U5 snRNP Biogenesis and the Spliceosome Active Site

Thi Hoang Duong Nguyen, Jade Li, Wojciech P. Galej, Hiroyuki Oshikane, Andrew J. Newman, and Kiyoshi Nagai

Inventory of Supplemental Information

Figure S1 shows the electron density map used for model building and the final electron density map. Related to Figure 1.

Figure S2 shows detailed structural alignments of human and yeast Brr2 structures and comparison of the level of conservation of the two helicase cassettes of yeast Brr2. Related to Figure 1.

Figure S3 shows multiple alignments of Brr2 and the Jab1/MPN domain of Prp8 from various organisms. Related to Figures 1 and 2.

Figure S4 shows the polar interaction network between the Jab1/MPN domain and N-terminal Sec63 unit of Brr2. Related to Figure 2.

Figure S5 shows the Jab1/MPN conformational change and crystal contact. Related to Figures 2 and 5.

Figure S6 shows a structural comparison of DNA-bound Hel308 and the N-terminal cassette of Brr2 and a model of the Brr2-Prp8 complex. Related to Figure 5.

Table S1 lists all the contact pairs between Brr2 and the Jab1/MPN domain. Related to Figure 2.

Table S2 lists all the RP mutations and their locations in the structure. Related to Figure 2.

Supplemental Experimental Procedures

Supplemental References

Figure S1. Initial electron density map used for model building and final electron density map. Related to Figure 1. (A) The unsharpened initial electron density map obtained from experimental phasing to 4.5 Å resolution followed by density modification and phase extension to 3.1 Å resolution (see Methods section). Molecular replacement solutions of the Jab1/MPN domain (PDB: 2OG4) (Pena *et al.*, 2007) and C-terminal Sec63 unit (C-Sec63) (PDB: 3HIB) (Zhang *et al.*, 2009) superimposed on the initial map. (B) Location of the Hel308 CHAINSAW model (Richards *et al.*, 2008) for the N- and C-terminal cassettes found in the initial density map by MOLREP (CCP4, 1994). (C) and (D) Initial electron density map as in (A) and (B) and $2mF_o\text{-}DF_c$ map of the same region at 1.5σ, respectively. (E) Final sigma-weighted $2mF_o\text{-}DF_c$ map of the active site (DEIH box, in magenta) of the N-terminal cassette shows good density for side-chains. The $2mF_o\text{-}DF_c$ map was sharpened by Refmac5 (Murshudov *et al.*, 2011) with regularised sharpening B-value of -48 $Å^2$.

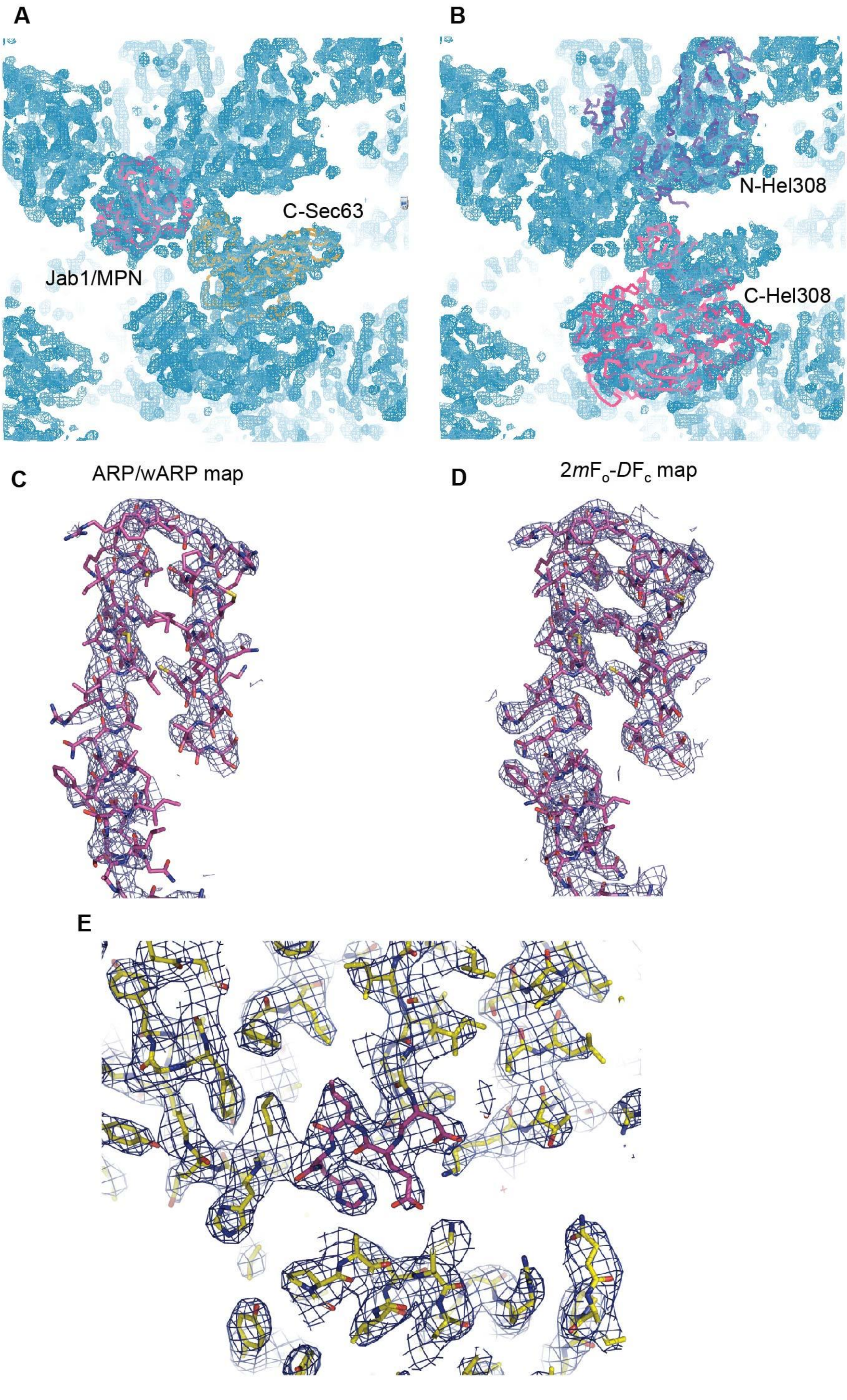
A
B
C-Sec63
Jab1/MPN
N-Hel308
C-Hel308
C
ARP/wARP map
D
2mFo-DFc map
E

Figure S2. Comparison of our yBrr2 structure (blue) and the published apo-hBrr2 (PDB: 4F91) (Santos *et al*., 2012). Related to Figure 1. (A) and (B) Superposition of the two RecA domains of N- and C-terminal cassettes, respectively. (C) and (D) Superposition of the remaining domains (domains 3-6) of the N- and C-terminal helicase cassettes, respectively. (E) and (F) Residue conservation of the N-terminal and C-terminal cassettes of Brr2, respectively calculated by ConSurf server (Ashkenazy *et al*., 2010). The N-terminal cassette shows greater conservation at the ATPase active site than the C-terminal cassette.

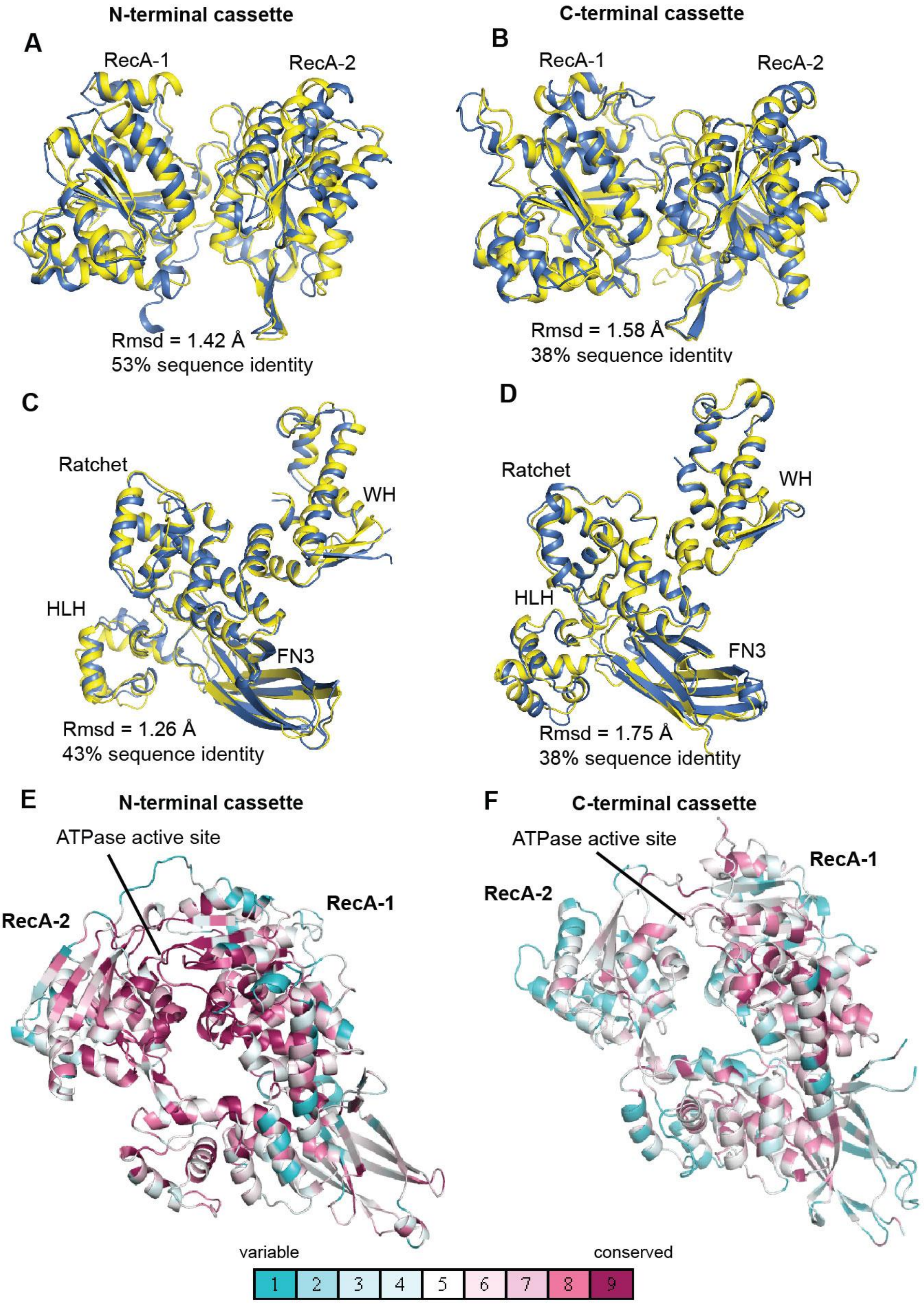
N-terminal cassette
C-terminal cassette
A
B
RecA-1
RecA-2
RecA-1
RecA-2
Rmsd = 1.42 Å
53% sequence identity
Rmsd = 1.58 Å
38% sequence identity
C
D
Ratchet
WH
HLH
FN3
Rmsd = 1.26 Å
43% sequence identity
Ratchet
WH
HLH
FN3
Rmsd = 1.75 Å
38% sequence identity
E
N-terminal cassette
F
C-terminal cassette
ATPase active site
RecA-2
RecA-1
ATPase active site
RecA-1
RecA-2
variable
conserved
1
2
3
4
5
6
7
8
9

Figure S3. Multiple sequence alignment of (A) Brr2 and (B) the Jab1/MPN domains from various organisms. Related to Figures 1 and 2. The two helicase cassettes are also aligned with each other in (A) with the helicase motifs indicated in gray blocks. Numbering refers to residues below the first digit of each number. Residues involved in yBrr2 and yJab1/MPN non-polar, polar and both interactions listed in Table S1 are indicated with black, red and cyan triangles, respectively.

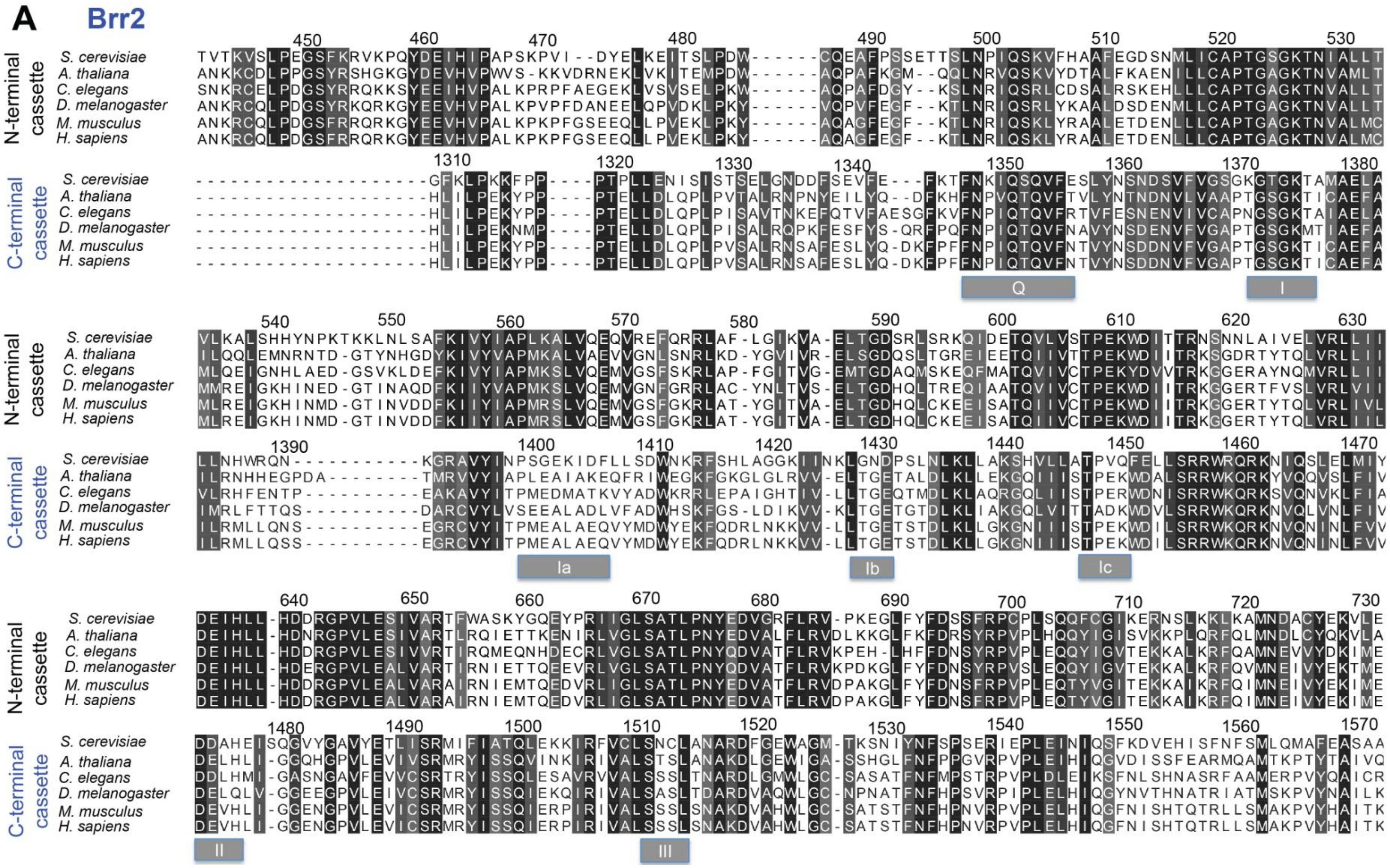
A
Brr2
N-terminal cassette
C-terminal cassette
S. cerevisiae
A. thaliana
C. elegans
D. melanogaster
M. musculus
H. sapiens
Q
I
Ia
Ib
Ic
II
III

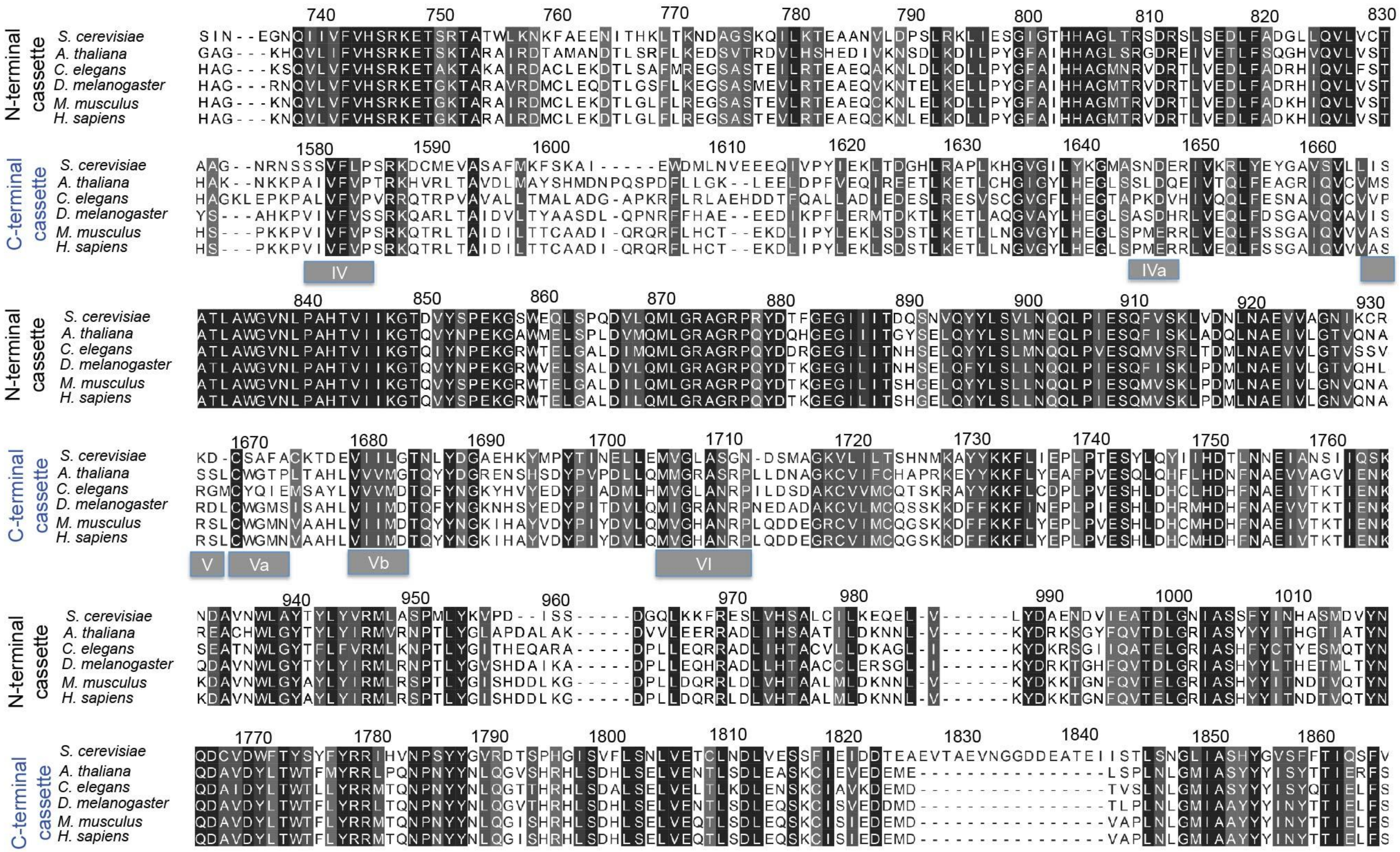

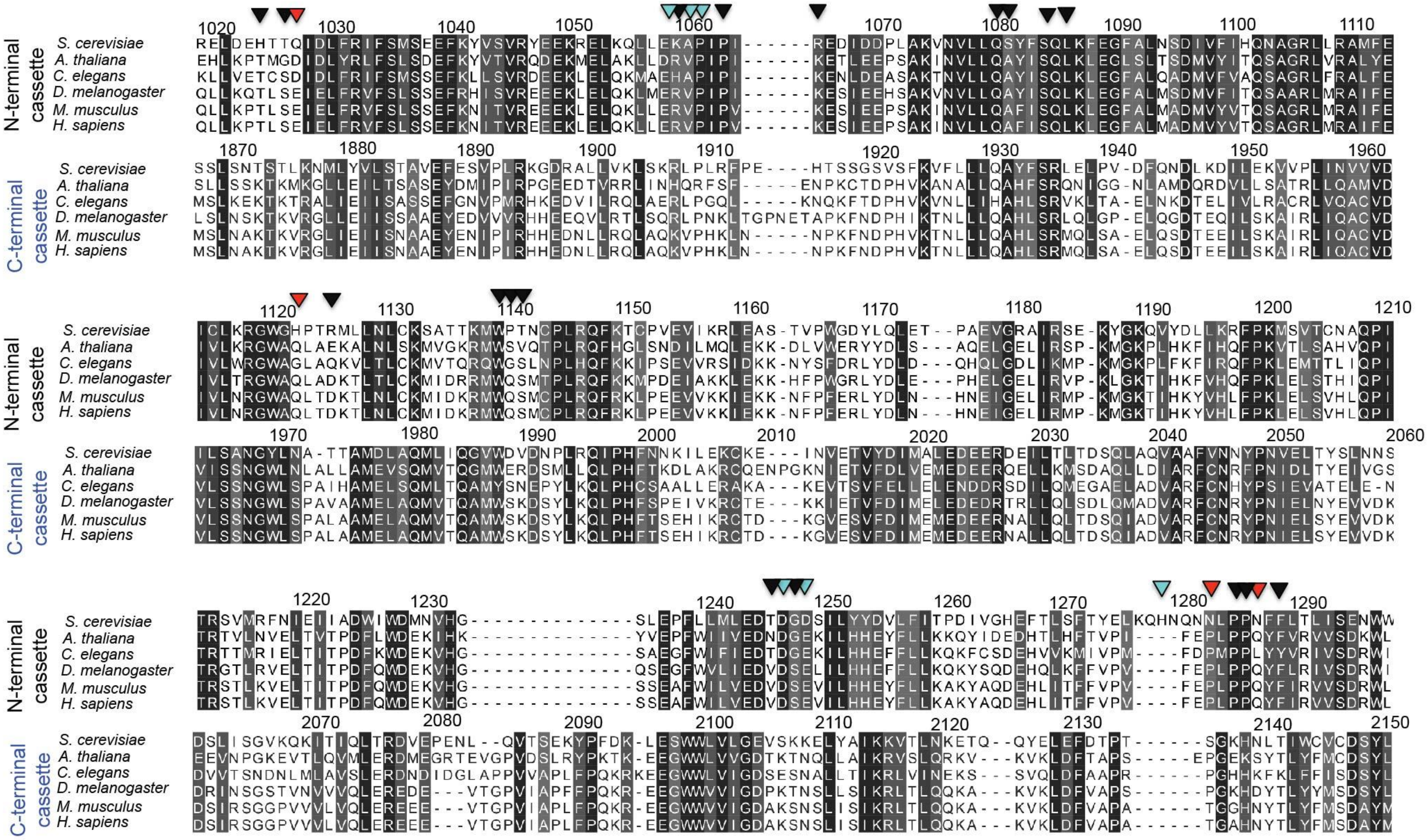
N-terminal cassette
C-terminal cassette
S. cerevisiae
A. thaliana
C. elegans
D. melanogaster
M. musculus
H. sapiens
1020 1030 1040 1050 1060 1070 1080 1090 1100 1110
1870 1880 1890 1900 1910 1920 1930 1940 1950 1960
1120 1130 1140 1150 1160 1170 1180 1190 1200 1210
1970 1980 1990 2000 2010 2020 2030 2040 2050 2060
1220 1230 1240 1250 1260 1270 1280 1290
2070 2080 2090 2100 2110 2120 2130 2140 2150

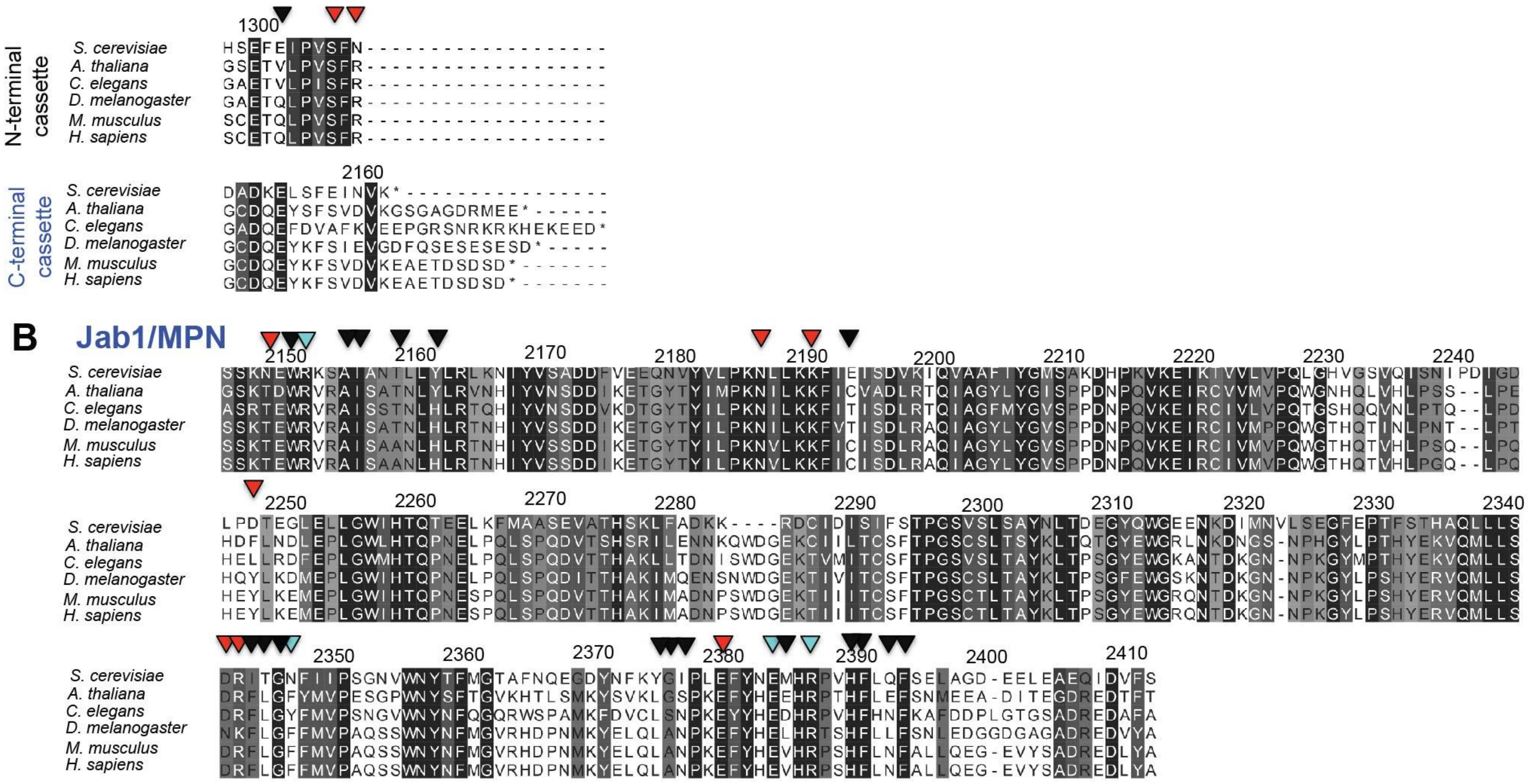

N-terminal cassette
1300
S. cerevisiae
A. thaliana
C. elegans
D. melanogaster
M. musculus
H. sapiens
C-terminal cassette
2160
B
Jab1/MPN
2150
2160
2170
2180
2190
2200
2210
2220
2230
2240
2250
2260
2270
2280
2290
2300
2310
2320
2330
2340
2350
2360
2370
2380
2390
2400
2410

Figure S4. Polar interactions between the N-terminal Sec63 unit (N-Sec63) and the Jab1/MPN domain. Related to Figure 2. (A) Highlighted interactions between the ratchet domain of Brr2 (yellow) and Jab1/MPN domain (magenta). Most of the highlighted interactions shown are polar except for the stacking interaction between *Y2163* and H1025. (B) The polar interaction network between the FN3 domain of Brr2 (red) and the Jab1/MPN domain (magenta). (C) Comparison of residues involved in polar interactions with the Jab1/MPN domain in the N-terminal Sec63 unit with the equivalent residues in the C-terminal Sec63 unit. Conserved and non-conserved residues between the two cassettes are coloured red and black, respectively. (D) Ni-NTA pull-down experiment showing that the Jab1/MPN domain does not bind to C-terminal Sec63 *in vitro*.

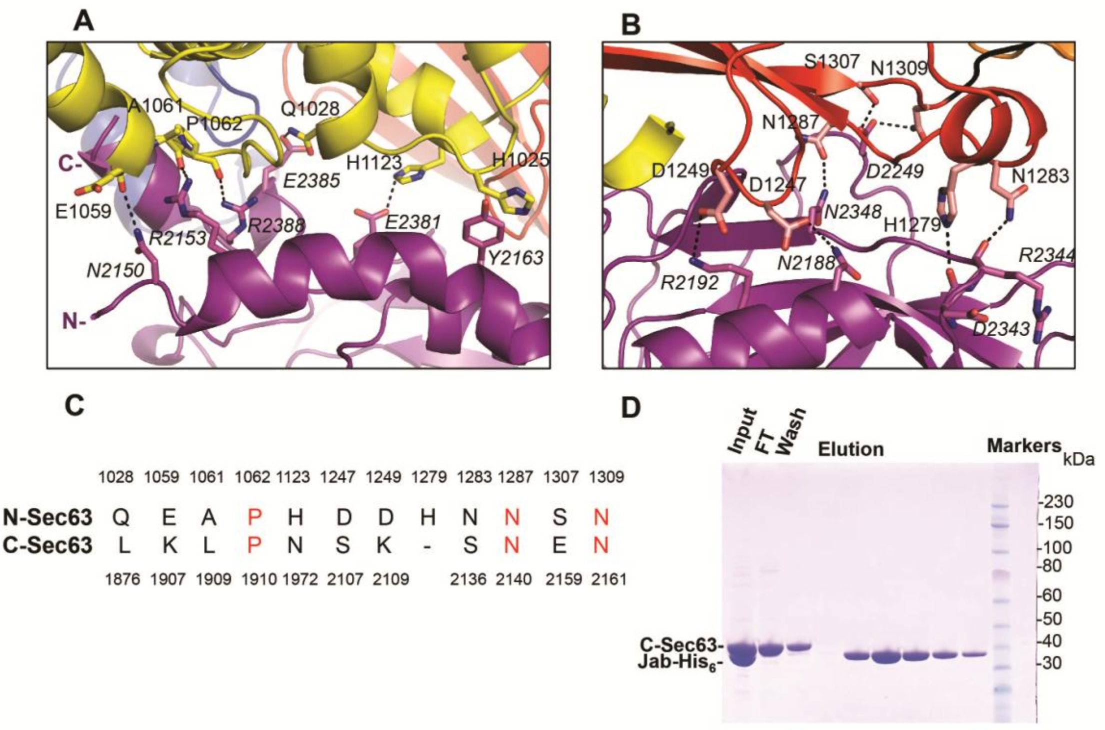
A
A1061
P1062
Q1028
C-
E2385
H1123
H1025
E1059
R2153
R2388
E2381
Y2163
N2150
N-
B
S1307
N1309
N1287
D1249
D2249
N1283
D1247
N2348
H1279
R2344
N2188
R2192
D2343
C
1028 1059 1061 1062 1123 1247 1249 1279 1283 1287 1307 1309
N-Sec63 Q E A P H D D H N N S N
C-Sec63 L K L P N S K - S N E N
1876 1907 1909 1910 1972 2107 2109 2136 2140 2159 2161
D
Input
FT
Wash
Elution
Markers
kDa
-230
-150
-100
-80
-60
-50
-40
-30
C-Sec63-
Jab-His6-

Figure S5. The Jab1/MPN domain conformational change and crystal contact. Related to Figures 2 and 5. (A) Superposition of the structures of free (grey, PDB: 2OG4, 3SBS, 3ZEF_A, 3ZEF_B, 4I43) and yBrr2-bound (magenta) Jab1/MPN domain with the N- and C-termini indicated (Pena *et al*., 2007; Galej *et al*., 2013; Weber *et al*., 2011). This shows that the major changes in the Jab1/MPN domain upon Brr2 binding are at its N- and C-termini. (B) Crystal contact between the Jab1/MPN domain and the C-terminal Sec63 unit of a symmetry-related molecule. *In vitro* pull-down assays (Figure S4D) show no binding of the Jab1/MPN domain to the C-terminal Sec63 unit. (C) The region of yeast Jab1/MPN domain (residues 2317-2413) corresponding to the human Jab1/MPN domain fragment (residues 2239-2335) used for the yeast two-hybrid studies (Pena *et al*., 2007) is highlighted in red, except for residues 2396-2413 which are missing in our structure. This fragment is not likely to fold into a stable domain. (D) Organization of the Jab1/MPN, RNaseH-like and RT/En domains of Prp8 by Aar2 (Galej *et al*., 2013). The Jab1/MPN domain (magenta) is fixed to the RT/En domain (grey) by the formation of a continuous β-sheet with the C-terminal tail of Aar2 (orange) and RNaseH-like domain (yellow) (PDB: 4I43). The removal of the C-terminal tail (23 residues) of Aar2 allows the Jab1/MPN to be free to interact with Brr2 (Figure 5A).

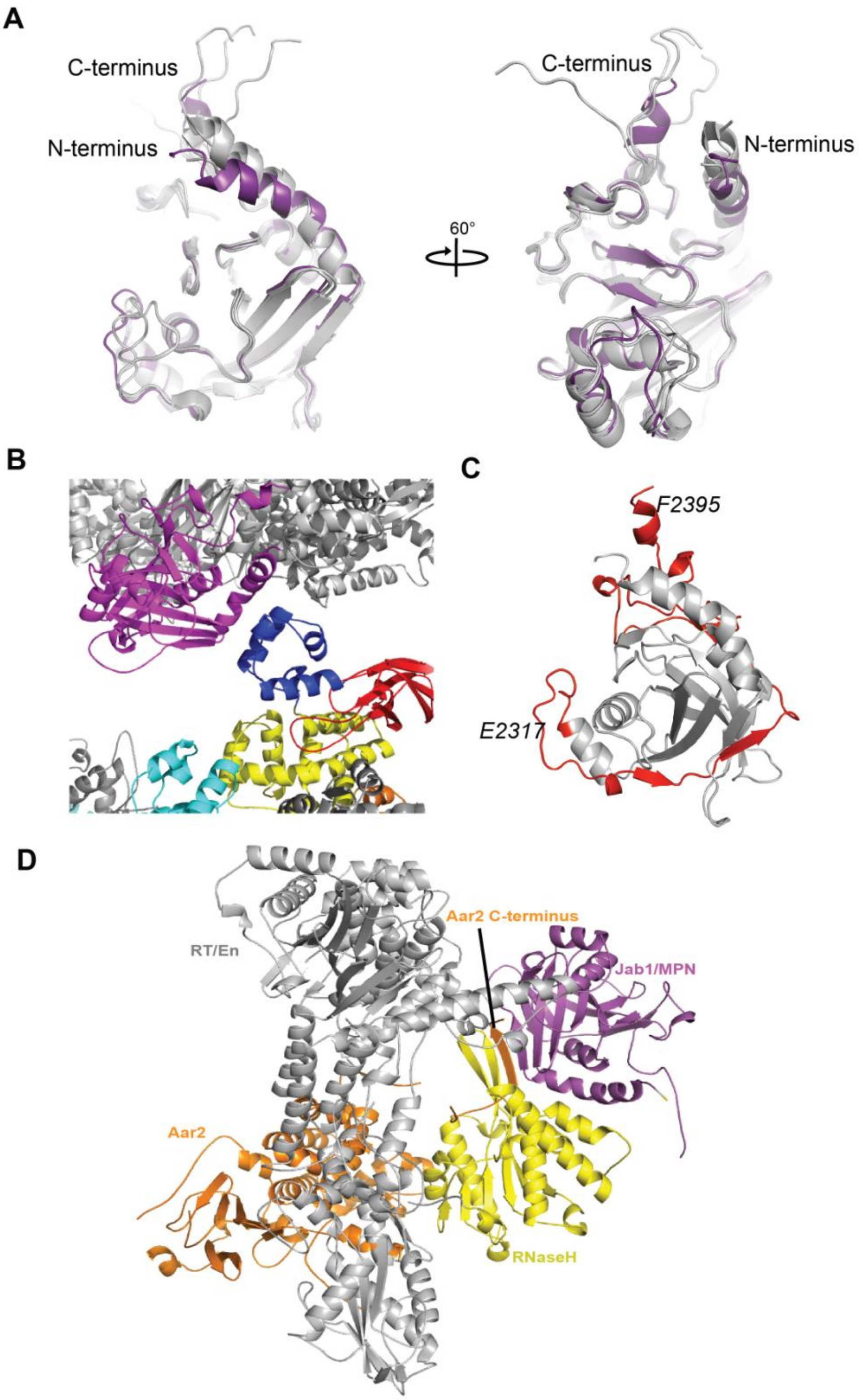
A
C-terminus
N-terminus
60°
C-terminus
N-terminus
B
C
F2395
E2317
D
Aar2 C-terminus
RT/En
Jab1/MPN
Aar2
RNaseH

Figure S6. Comparison of DNA-bound Hel308 and Brr2 N-terminal cassette structures and a model of the Brr2-Prp8 complex. Related to Figure 5. (A) DNA-bound Hel308 structure (PDB: 2PR6) shows the emergent 3'-strand (gold) traveling down the channel formed by the RecA-1 (grey), WH (orange) and ratchet (yellow) domains and along the HLH (blue) domain (Büttner *et al*., 2007). (B) Brr2 N-terminal cassette in the same orientation as Hel308 structure in (B) and the modeled DNA duplex from the Hel308 structure. This shows that the cleft between the RecA-2 (cyan), ratchet and HLH domains in Brr2 is wider than that of the Hel308 structure in (A).

(C) Brr2 may form an additional wall of the active site cavity together with the RT/En domain of Prp8. DNA molecule from the Hel308:DNA complex (PDB: 2P6R) was placed into the N-terminal helicase cassette of Brr2 by overlaying the corresponding domains 1-5 (RecA-1, RecA-2, WH, ratchet and HLH) of Hel308 and Brr2. Suppressors for U4-cs1 and *brr2-1* (yellow spheres) map on one surface of Prp8 indicating likely Brr2 binding interface (Galej *et al*., 2013). The Brr2-Jab1/MPN domain structure was docked to the Prp8 RT/En domain (PDB: 4I43) based on the surface complementarity and location of U4-cs1 *and brr2-1* mutations. The RNaseH-like domain (U4-cs1 suppressors are labeled as green spheres) is linked to the En domain with a short linker and to the Jab1/MPN domain with a 70-residue linker (dotted line). The position of the RNaseH-like domain with respect to the RT/En domain is shown as in the Prp8-Aar2 complex (Galej *et al*., 2013). When Aar2 is replaced by Brr2 in the active spliceosome, the RNaseH-like domain is only constrained by the linkers and hence its position can be modulated by the conformational change of the active site RNA. The long linker peptide may act as reins to alter the position of the RNaseH-like domain in response to its interaction with other factors. Partially unwound DNA (yellow), represents fragment of U4/U6 snRNA, which is being unwound by Brr2 (different shades of blue) and fed into the active site cavity formed by Prp8 (different shades of red) and Brr2.

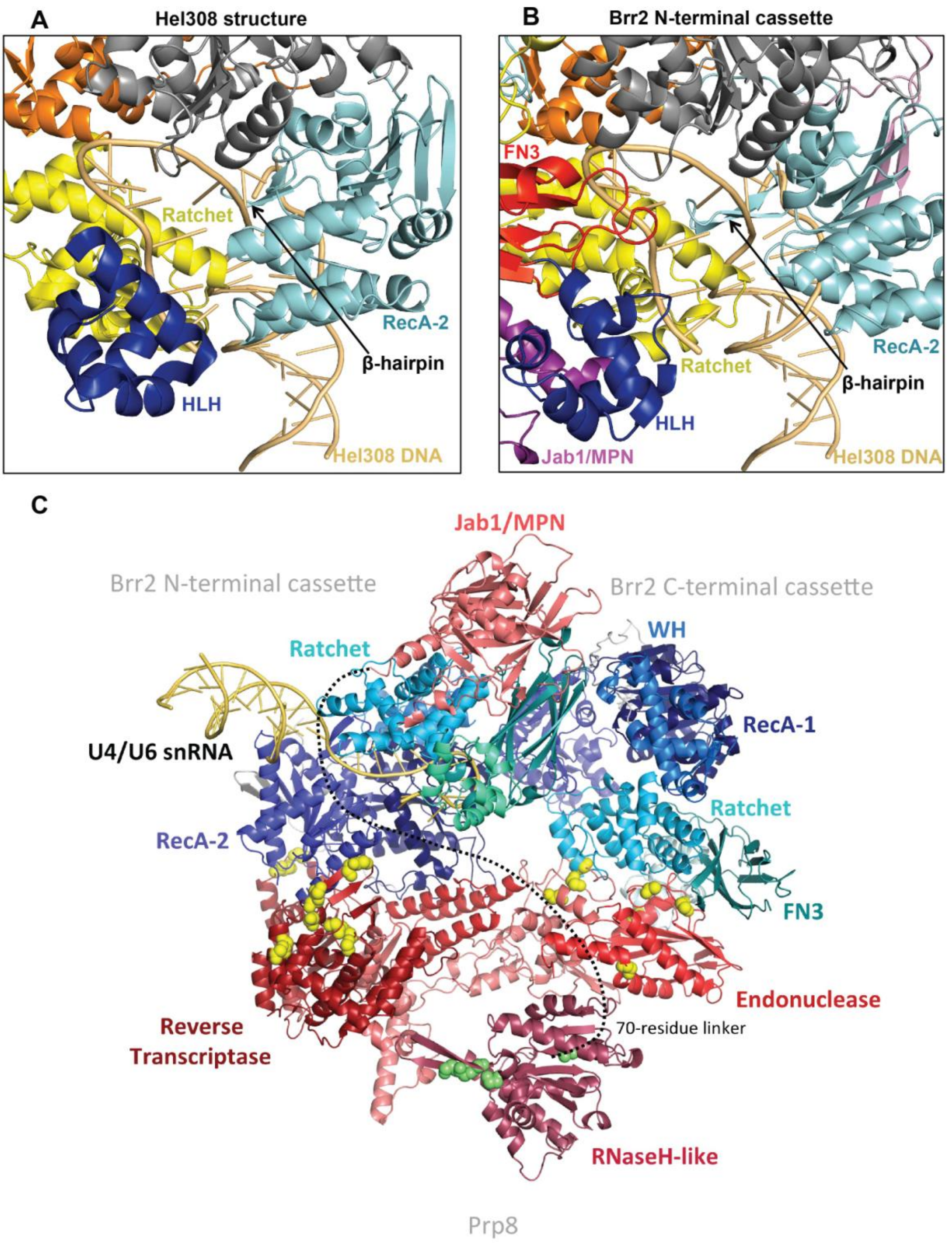
A
Hel308 structure
Ratchet
RecA-2
β-hairpin
HLH
Hel308 DNA
B
Brr2 N-terminal cassette
FN3
Ratchet
RecA-2
β-hairpin
HLH
Jab1/MPN
Hel308 DNA
C
Jab1/MPN
Brr2 N-terminal cassette
Brr2 C-terminal cassette
WH
Ratchet
RecA-1
U4/U6 snRNA
Ratchet
RecA-2
FN3
Endonuclease
Reverse Transcriptase
70-residue linker
RNaseH-like
Prp8

Table S1. Brr2-Jab1/MPN contact pairs*. Related to Figure 2.

Brr2 domains	Brr2 residues	Jab1/MPN residues
Ratchet domain	1023 Asp	2163 Tyr
	1025 His	2163 Tyr
	1027 Thr	2160 Thr
	1028 Gln	2385 Glu
	1029 Ile	2156 Ala
	1029 Ile	2157 Ile
	1057 Leu	2153 Arg
	1057 Leu	2395 Phe
	1059 Glu	2153 Arg
	1059 Glu	2150 Asn
	1060 Lys	2395 Phe
	1060 Lys	2394 Gln
	1061 Ala	2153 Arg
	1061 Ala	2395 Phe
	1062 Pro	2391 His
	1062 Pro	2392 Phe
	1062 Pro	2395 Phe
	1062 Pro	2152 Trp
	1062 Pro	2388 Arg
	1063 Ile	2388 Arg
	1063 Ile	2393 Phe
	1064 Pro	2153 Arg
	1064 Pro	2157 Ile
	1066 Arg	2157 Ile
	1081 Gln	2395 Phe
	1082 Ser	2395 Phe
	1085 Ser	2395 Phe
	1087 Leu	2395 Phe
	1123 His	2381 Glu
	1126 Arg	2378 Ile
	1140 Trp	2392 Phe
	1140 Trp	2385 Glu
	1141 Pro	2385 Glu
	1142 Thr	2385 Glu
	1142 Thr	2386 Met
	1142 Thr	2392 Phe
FN3 domain	1244 Glu	2378 Ile
	1246 Thr	2347 Gly
	1246 Thr	2348 Asn
	1247 Asp	2378 Ile
	1247 Asp	2188 Asn
	1248 Gly	2378 Ile
	1249 Asp	2195 Glu
	1249 Asp	2192 Lys
	1279 His	2343 Asp
	1279 His	2346 Thr
	1283 Asn	2344 Arg

1283 Asn	2345 Ile
1285 Pro	2346 Thr
1286 Pro	2347 Gly
1286 Pro	2348 Asn
1287 Asn	2348 Asn
1289 Phe	2377 Gly
1303 Glu	2377 Gly
1303 Glu	2378 Ile
1305 Pro	2376 Tyr
1307 Ser	2249 Asp
1309 Asp	2249 Asp

* Pairs of contact atoms between Brr2 and the Jab1/MPN domain were identified by the CCP4 program CONTACT, using a cut-off distance of 4.5 Å. Residues containing the contact atoms are listed. Red lettering indicates H-bonding. Shading under Jab1/MPN residues indicates whether the residue is from the N-terminal part (blue) or C-terminal part (orange) of that domain. This table and Figure S4 show that in contacts involving the Jab1/MPN domain polar interactions are interspersed among van der Waals interactions, whereas with the FN3 domain polar interactions are extensive.

Table S2. Retinitis Pigmentosa type 13 mutations[a]. Related to Figure 2.

RP mutation in human	Yeast residue	Effect of yeast mutation[b]	Location in the structure
P2301T	P2379	NR	Stabilises loop at the interface
F2304L	F2382	48% binding	Stabilises loop at the interface
H2309P	H2387	0.3 % binding	Supporting helix at the interface
H2309R	H2387	0.8 % binding	Supporting helix at the interface
R2310G	R2388	2% binding	Hydrogen bonded to the main chain CO of Pro1062 of rachet domain
R2310K	R2388	1% binding	Hydrogen bonded to the main chain CO of Pro1062 of rachet domain
F2314L	F2392	NR	Packed against hydrophobic residues in rachet domain
Q2321TER	A2399	NR	Not visible
E2331, 1 basepair deletion	I2409	NR	Not visible
Y2334N	F2412	NR	Not visible

[a] McKie *et al*. (2001); Martinez-Gimeno *et al*., (2003); De Erkenez *et al*., (2002); Kondo *et al*., (2003); van Lith-Verhoeven *et al*., (2002).
[b] Binding was assayed using Brr2 and the GST-tagged fragment containing the RNaseH and Jab1/MPN domain (Maeder *et al*., 2009); NR, not reported.

Supplementary Methods

Ni-NTA pull-down experiment of the Jab1/MPN domain and C-terminal Sec63

His_6-Brr2$^{1863\text{-}2163}$ (His_6-C-Sec63) was expressed using pET28a vector (Novagene) in *E. coli* strain BL21(DE3) pLysS and purified by a Ni-NTA column using the same procedures as the Prp8 fragments. The His_6 tag was cleaved from C-Sec63 fragment by thrombin overnight at room temperature before passing the mixture through a Ni-NTA column to remove uncleaved protein and the His_6 tag. The protein was dialyzed against B150 (20 mM Tris HCl pH 8.0, 150 mM NaCl and 10 mM β-mercaptoethanol) and further purified on a MonoQ column pre-equilibrated with B150 using NaCl gradient from 150 to 1 M. His_6-tagged Jab1/MPN domain (40 µM) was mixed with a two-fold molar excess of untagged C-Sec63 overnight in Ni250W buffer (20 mM Tris HCl pH 8.0, 250 mM NaCl, 10 mM imidazole and 10 mM β-mercaptoethanol). The mixture was incubated with Ni-NTA resin for 3 hours at 4 °C. The resin was washed with Ni250W buffer and the proteins were eluted with Ni250E buffer (20 mM Tris HCl pH 8.0, 250 mM NaCl, 250 mM imidazole and 10 mM β-mercaptoethanol). The input, flow-through, wash and elution fractions were analysed by SDS-PAGE gel (Figure S4D).

Supplementary references

Ashkenazy, H., Erez, E., Martz, E., Pupko, T. and Ben-Tal, N. (2010). ConSurf 2010 calculating evolutionary conservation in sequence and structure of proteins and nucleic acids. Nucl. Acids Res. *38* (Web Server issue): W529–W533.

De Erkenez, A. C., Berson, E. L., and Dryja, T. P. (2002). Novel mutations in the PRPC8 gene, encoding a pre-mRNA splicing factor in patients with autosomal dominant Retinitis Pigmentosa. ARVO 2002: (online abstract).

Martinez-Gimeno, M. *et al*. (2003). Mutations in the pre-mRNA splicing-factor genes PRPF3, PRPF8, and PRPF31 in Spanish families with autosomal dominant retinitis pigmentosa. Invest. Ophthalmol. Vis. Sci. *44*, 2171–2177.

Kondo, H., Tahira, T., Mizota, A., Adachi-Usami, E., Oshima, K., and Hayashi, K. (2003). Diagnosis of autosomal dominant retinitis pig-mentosa by linkage-based exclusion screening with multiple locus-specific microsatellite markers. Invest. Ophthalmol. Vis. Sci. *44*, 1275–1281.

van Lith-Verhoeven, J. J., van der Velde-Visser, S. D., Sohocki, M. M., Deutman, A. F., Brink, H. M., Cremers, F. P., and Hoyng, C. B. (2002). Clinical characterization, linkage analysis, and PRPC8 mutation analysis of a family with autosomal dominant retinitis pigmentosa type 13 (RP13). Ophthalmic Genet. *23*, 1–12.
